# COVID-19-Associated Middle Ear Myoclonus in a 10-Year-Old Male

**DOI:** 10.7759/cureus.24550

**Published:** 2022-04-28

**Authors:** Gabrielle LeBlanc, Patricia Blanco

**Affiliations:** 1 Medicine, Florida State University College of Medicine, Tallahassee, USA; 2 Pediatrics, Johns Hopkins All Children’s Hospital, Sarasota, USA

**Keywords:** covid-19, middle ear myoclonus, objective tinnitus, stapedius, tensor tympani

## Abstract

Middle ear myoclonus is a rare condition attributed to abnormal, repetitive contractions of the middle ear muscles including the tensor tympani and/or stapedius muscles. This condition generates objective tinnitus that is characterized by a “clicking” noise that is audible to both the patient and an outside observer. No specific pathophysiologic process has been identified as the cause of middle ear myoclonus, making its diagnosis and treatment challenging. In this report, we present a presumptive case of COVID-19-associated middle ear myoclonus in a 10-year-old male.

## Introduction

Tinnitus is defined as the perception of sound in the absence of a true external auditory stimulus [1]. The differential to consider when evaluating tinnitus is vast, making it rather difficult to diagnose. However, the characteristics of tinnitus, including quality, location, pitch, and variability, may point to its etiology. Tinnitus can be subjective or objective. Subjective tinnitus is only audible to the patient, while objective tinnitus is audible to the patient as well as an outside observer [2]. This observer can appreciate the tinnitus with their naked ear or through auscultation of the head and/or neck structures located close to the ear with a stethoscope.

Although rare, cases of objective tinnitus have been reported to develop due to repetitive abnormal rhythmic contractions of middle ear muscles, including the stapedius and tensor tympani, and palatal muscles [3]. Specifically, tensor tympani myoclonic contractions are characterized as “clicking,” while “buzzing” is a characteristic of stapedial contractions [4]. Tinnitus of this type is known as middle ear myoclonus.

Middle ear myoclonus accounts for only 1.5% of new tinnitus cases. With such a small number of diagnoses, data and understanding of this condition are significantly limited with most of the published literature taking the form of case reports and case series [5,6]. The definitive etiology of tinnitus produced by middle ear myoclonus is unknown. There are, however, many theories as to how this condition is produced. A variety of causes including brain tumors such as acoustic neuromas, vascular deformities, trauma, anxiety, demyelinating disease, and viral infections have been implicated [7]. Furthermore, several cases of middle ear myoclonus have been attributed to aberrant activity or irritability of the facial and trigeminal nerves and nuclei, which control the actions of both the tensor tympani and stapedius muscles [4].

In this report, we present the case of a 10-year-old male diagnosed with middle ear myoclonus after a presumed COVID-19 viral infection.

## Case presentation

A 10-year-old male, with a past medical history significant for autism and attention deficit hyperactivity disorder (ADHD), presented to the outpatient pediatric clinic reporting a popping, clicking noise that had been present in both of his ears for approximately five months. Upon entrance to the patient’s room, the child eagerly ran to the medical student, exclaiming, “Listen to my ears!” Disregarding personal space, he pulled the medical student’s ear to his own and instructed her to listen. A steady, rhythmic audible clicking noise was heard by the examiner in both ears. The clicking noise could be described as medium-to-high pitch, constant and rhythmic in nature, and did not appear to correlate with the patient’s heartbeat.

Upon physical examination, vital signs were normal, with a blood pressure of 91/51 mmHg, pulse of 94 beats per minute, and temperature of 97.2°F. Further examination showed that the bilateral ear canals were normal in both size and shape and free of any discharge. Right and left tympanic membranes were pearly white and clear with no evidence of perforation or scarring. The right and left middle ears were free of effusion or infection. Hearing appeared to be intact. A nasal examination revealed mild congestion of both nares. The oral cavity, oropharynx, hypopharynx, and neck were grossly normal. No palatal muscle movement was observed. The jaw was non-tender to palpation with a full range of motion. A neurologic examination showed cranial nerves II-XII intact. The remainder of the physical examination was unremarkable.

The patient and his mother reported that the sound had been progressively worsening. The noise became louder, more constant in nature, and increasingly bothersome to the child. The patient stated that the clicking sound improved when submerged underwater and disappeared during sleep or when the patient held his breath. Additional symptoms included difficulty hearing, sinus pressure, bilateral nasal congestion, and sore throat. The patient denied jaw pain, vertigo, dizziness, headache, cough, reflux, or changes in vision. Additionally, the patient’s mother denied any previous ear disease, ear trauma, head injuries, or exposure to loud noises.

The patient’s mother reported that the child’s symptoms began shortly after a protracted respiratory illness in January 2021, which was presumed to be COVID-19. Other members of the household, including the patient’s mother, father, and siblings, were ill at the same time, and all had tested positive for the virus. Because of the child’s sensory issues and sensitivity to nasopharyngeal swabbing, the patient’s mother opted not to have him tested for active infection. Additionally, no antibody testing was performed. The family members all achieved complete resolution of their symptoms; however, the patient continued to display intermittent nasal congestion that worsened over the next several weeks. He was first seen in the pediatric clinic three months after the appearance of symptoms, which he originally described as ear discomfort, cough, and sinus congestion. The patient was diagnosed with acute sinusitis and was prescribed oral amoxicillin for treatment, which he refused to take due to taste.

The patient returned to the clinic three weeks later with worsening sinus congestion and decreased hearing in both ears. He was started on fluticasone propionate nasal spray and was referred to a pediatric ear, nose, and throat (ENT) specialist for further evaluation. When the patient visited the ENT two weeks later, he was able to describe the ear discomfort he had been experiencing as a “clicking” noise. A hearing test was performed, and tympanograms were obtained for both ears (Table 1); all results were normal, and the child was diagnosed with middle ear myoclonus. He was continued on fluticasone for treatment. Follow-up with ENT one month later showed no improvement. The ENT ordered a magnetic resonance angiogram (MRA) of the brain, which was not completed.

Upon diagnosis of middle ear myoclonus, initial treatment focused on attempting to treat the ongoing upper respiratory symptoms in hopes that the patient’s tinnitus would resolve as well. A regimen of medications including fluticasone nasal spray as needed, cetirizine-pseudoephedrine in the morning, and diphenhydramine 25 mg nightly was started in an attempt to improve the patient’s upper respiratory symptoms. However, after follow-up roughly four months later, there had still been no improvement in the patient’s tinnitus despite the resolution of his upper respiratory symptoms. The patient’s mother reported that the patient was learning to live with the tinnitus; however, they were open to other treatment options.

**Table 1:** Tympanometry results *Normal range of values describing external ear canal volume, compliance, and pressure. ^a^Type A tympanograms are considered normal. ^b^Compliance refers to tympanic membrane movement or how well the middle ear responds to sound. ^c^Pressure refers to the pressure of air contained within the middle ear.

Tympanometry				
	Type^a^	External ear canal volume (mL) (0.5-1.5)*	Compliance^b^ (mL) (0.3-1.5)*	Pressure^c^ (daPa) (-200-+50)*
Right ear	A	1.29	0.52	-16
Left ear	A	1.25	0.52	-23

## Discussion

Initially, many etiologies were considered in determining this patient’s final diagnosis, many of which are illustrated in Table 2 [8-10]. It is important to recognize the type of tinnitus presenting in this case and its manifestation in the patient. Tinnitus can be subjective or objective. Subjective tinnitus is only audible to the patient, while objective tinnitus is audible to the patient as well as an outside observer [2]. This patient presented with clear objective tinnitus evident by the ability of outside observers to appreciate the sound.

**Table 2:** Differential diagnoses for subjective and objective tinnitus

Differential diagnoses	Etiologies
Subjective tinnitus	
Otologic	Presbycusis, noise-induced hearing loss, congenital hearing loss, otosclerosis, cholesteatoma, otitis, impacted cerumen, Meniere’s disease, bone disease (osteogenesis imperfecta, Paget’s disease of bone)
Neurologic	Multiple sclerosis, Chiari malformation, idiopathic intracranial hypertension, vestibular migraine, stroke, vestibular schwannoma, cerebellopontine angle tumors
Infectious	Acute and chronic otitis media, Lyme disease, meningitis, neurosyphilis, rubella, measles, cytomegalovirus (CMV), fungal, viral, and bacterial infectious or inflammatory processes
Somatic and traumatic	Head and neck injuries, whiplash, temporomandibular joint dysfunction, cerumen removal, dental disorders
Drug-related	Antibiotics, salicylates, loop diuretics, platinum-based chemotherapy, aminoglycosides, nonsteroidal anti-inflammatory drugs (NSAIDs)
Psychogenic	Anxiety, depression, fibromyalgia
Objective tinnitus	
Vascular (pulsatile)	Arteriovenous malformation, vascular tumors, carotid atherosclerosis, stenosis, dissection, or tortuosity, arterial bruit, venous hum, valvular heart disease, small vessel disease, diabetic vasculopathy, high cardiac output states (anemia, sickle cell, pregnancy)
Muscular or anatomical (nonpulsatile)	Palatal myoclonus, tensor tympani myoclonus, stapedius myoclonus, patulous eustachian tube

Originally, an otologic origin of hearing loss and tinnitus caused by a chronic otitis media was considered. However, no cerumen impaction, auditory canal swelling, tympanic membrane injury, middle ear fluid, or otosclerosis were appreciated on physical examination [9]. These etiologies are known to produce subjective tinnitus, which was not consistent with this patient’s presentation. Other causes of subjective tinnitus that were excluded were head trauma or exposure to loud noise, all of which were not applicable based on the patient’s history.

Other possible otologic causes included Meniere’s disease and acoustic neuroma. The patient, however, did not present with concurrent symptoms such as a sensation of fullness in the ears, dizziness, or vertigo. Additionally, bilateral tympanograms were normal. Psychological causes stemming from the patient’s autism, ADHD, and overall sensory difficulties seemed unlikely as the patient’s symptoms were audible to the examiner.

In further investigation of the differential diagnosis for objective tinnitus, arterial and venous vascular origins including, but not limited to, glomus tumors, congenital arteriovenous malformations, and anemia were considered [11]. However, objective tinnitus produced from a vascular abnormality presents as pulsatile tinnitus that coordinates with the patient’s pulse and is usually worse at nighttime [9]. This patient’s ear clicking did not synchronize with his heartbeat and did not worsen at nighttime; rather, it disappeared when the patient was asleep.

The neurologic causes of objective tinnitus including muscular origins due to middle ear myoclonus were investigated. Although rare, this condition develops due to abnormal rhythmic contractions of middle ear muscles, including the stapedius and tensor tympani, and palatal muscles [3]. Specifically, tensor tympani myoclonic contractions are characterized as “clicking,” while “buzzing” is a characteristic of stapedial contractions [4]. This type of myoclonus is elicited by various agents, including viral infections [7]. Based on the patient’s presentation with nonpulsatile clicking tinnitus with the onset of symptoms at the same time as prolonged COVID-19 recovery, this patient was ultimately diagnosed with middle ear myoclonus. The development of this objective tinnitus from middle ear myoclonus is credited to COVID-19 infection, which likely induced damage or inflammation of the facial and trigeminal nerves or nuclei [4,12].

In the wake of a pandemic created by a novel coronavirus, knowledge of how this virus affects individuals has been changing and expanding rapidly over time. Research shows that COVID-19 infection is closely linked to various otolaryngological symptoms, including anosmia, ageusia, tinnitus, sudden deafness, hoarseness, and Bell’s palsy [13]. In research produced by cadaveric studies performed at Johns Hopkins, scientists were able to isolate the virus from both mastoid air cells and middle ears of patients, confirming that SARS-CoV-2 can infect these areas of the body, leading to various sensory deficits [14]. Additionally, in patients diagnosed with COVID-19 experiencing audiovestibular symptoms, researchers examined human inner ear tissue, demonstrating that cells of this tissue possess the molecular machinery necessary for SARS-CoV-2 viral entry and infection [15]. Viral invasion and damage of inner ear structures can result in the inflammation of vital inner ear structures and neuroinflammation, leading to long-term audiovestibular damage and symptoms. In this patient, the diagnosis of middle ear myoclonus most closely coincides with COVID-19 viral infection targeting the virus as the most likely etiology.

Middle ear myoclonus has been treated with conservative methods, including muscle relaxants, anticonvulsants, zygomatic pressure maneuvers, and botulinum toxin injections to the muscles of the middle ear. However, if patients fail conservative therapy, more permanent treatment with surgical transection of the stapedius and tensor tympani muscle tendons has produced excellent therapeutic results [12].

## Conclusions

Middle ear myoclonus continues to be a rare condition manifesting as objective tinnitus due to abnormal myoclonic contractions of the inner ear muscles. Middle ear myoclonus has been attributed to a wide variety of acoustogenic, myogenic, and psychogenic etiologies. It has also been attributed to viral infection, which was seen in the patient described in this case who experienced COVID-19 infection prior to developing symptoms. With SARS-CoV-2 viral infection being closely linked to otolaryngological symptoms, further research is needed to better describe the mechanism leading to these effects. Middle ear myoclonus can be treated by various methods, including conservative management with medications or more extensive surgical management. This case adds to the already limited body of literature describing middle ear myoclonus, especially in the pediatric population.
